# Angiostrongyliasis: A Changing Scenario?

**DOI:** 10.3390/pathogens12101214

**Published:** 2023-10-03

**Authors:** Fabrizio Bruschi

**Affiliations:** Department of Translational Research, New Technologies in Medicine and Surgery, Università di Pisa, 56126 Pisa, Italy; fabrizio.bruschi@unipi.it

Angiostrongyliasis is a parasitic disease caused by larvae of the genus *Angiostrongylus*, with *Angiostrongylus cantonensis* and *Angiostrongylus costaricensis* being the two main species causing diseases in humans [1]. 

According to Cowie et al. [2], it is an emerging but still neglected disease, although it is not yet present in the WHO list of Neglected Tropical Diseases (see: https://www.who.int/health-topics/neglected-tropical-diseases#tab=tab_1, accessed on 1 August 2023).

Other species belonging to the genus *Angiostrongylus* include *Angiostrongylus vasorum,* which infect domestic dogs, and *Angiostrongylus chabaudi*, *Angiostrongylus daskalovi* and *Angiostrongylus dujardini*, which mainly infect wild animals, but their possible zoonotic role is unknown [3].

Adult worms of *A. cantonensis* reside in the pulmonary arteries and right ventricle of the definitive host (i.e., rats). Once fecundated, females lay eggs, which hatch in the terminal branches of the pulmonary arteries, from which L_1_ larvae are released. These migrate to the pharynx, where they are swallowed and pass through the digestive tract to be eliminated in the stool, which will contaminate places where they can penetrate or be ingested by a gastropod (either snails or slugs), the intermediate host. After two molts, L_3_ larvae become infective to mammals, including humans. After the infected gastropod is ingested by the definitive host, L_3_ larvae migrate to the brain and develop into young adults, which will return to the venous system and then to the pulmonary arteries, where they reach sexual maturity. 

A variety of paratenic (only transporting) hosts (freshwater shrimp, crabs, and mollusks) can also harbor the L_3_ infective larvae, which can develop further once the paratenic host is ingested by rodents.

The life cycle is shown at the following link: https://www.cdc.gov/dpdx/angiostrongyliasis_can/index.html, accessed on 1 August 2023.

Human infections are acquired by the ingestion of raw or undercooked snails or slugs or vegetables containing the infective larvae. Infection can also be acquired by eating raw products containing a small snail or slug, or part of one. In humans, larvae migrate to the brain (or less frequently to the lungs), where the worms, unable to reach full maturation, ultimately die. Human angiostrongyliasis is widely distributed worldwide. It is endemic in tropics and subtropical areas in South Asia, the Pacific Islands, Australia, and the Caribbean islands, with sporadic cases also occurring in temperate climatic zones. In Europe, *A. cantonensis* has been recorded in islands south of Europe, and was recorded for the first time in the Canary Islands and, more recently, in the Balearic Islands, although there is limited evidence of zoonotic transmission to date [3].

In humans, the disease occurs in two clinical forms: *Angiostrongylus* eosinophilic meningitis (AEM) and ocular.

New information is provided by Pandian et al. [4] and Galan-Puchades et al. [5] regarding the circulation of *A. cantonensis* in the Indian subcontinent (Pakistan, India, Maldives, Nepal, Sri Lanka, Bangladesh, and Bhutan) and continental Spain, respectively.

In the Indian report [4], the first in this geographical region, in a period lasting over 50 years, 45 human cases were reported: 33 cases of AEM and 12 cases with ocular involvement, 1 combined case and 1 unspecified case. The prevalence of ocular disease was higher than in other epidemiological studies, for example, in Thailand or China [6,7], suggesting the possible involvement of other *Angiostrongylus* species or *A. cantonensis* genotypes in the Indian sub-continent.

What is interesting in the Indian report is the fact that 22 patients affected by AEM reported a history of consumption of raw monitor lizard (*Varanus* spp.) tissues, particularly in the Keratala and Kerala provinces. Since these animals can contain a high concentration of L_3_ larvae, being at the apex of the food chain, the authors suggest considering them as effective sentinals in the Indian subcontinent, in addition to rodent and mollusk hosts. However, unfortunately, according to the authors, no data exist on the prevalence of *A. cantonensis* infection in saurian reptiles in the Indian subcontinent. 

Monitor lizards have also been recognised as a source of contaminated food in an angiostrongyliasis cluster (seven cases) in Lao People’s Democratic Republic [8].

The limit of the Indian study is that a molecular analysis aiming to identify the *Angiostrongylus* species was carried out in only two cases where *A. cantonensis* was ascertained; in the other cases, the presence of other *Angiostrongylus* species cannot be ruled out. However, if molecular analyses can distinguish three related species, namely *A. cantonensis*, *A. malaysiensis*, and *A. mackerrasae* [9], only the first species can be considered zoonotic based on current knowledge. 

The report from Spain [5] updates the epidemiological situation after the first observation of the parasite in continental Europe, in the province of Valencia (Spain), in rats trapped in the sewer system [10], although an enigmatic autochtonous case, which was only serologically proven, was previously described in France [11].

Galan-Puchades and coll. found the parasite in 8 (5 *R. norvegicus* and 3 *R. rattus*) out of 94 analyzed rats. The highest prevalence of infection (20%) was found in rats trapped in the orchards surrounding the city of Valencia, where snails and slugs are plentiful.

The Maxent program was used to develop an ecological niche model. Regarding the possible future distribution of *A. cantonensis,* in consideration of climate changes, a diffusion is predicted to occur by the 2050s from the equatorial areas to Northern regions of the globe [12]. 

The findings of rat lungworms in the Balearic Island of Mallorca and Valencia are consistent with this prediction of the future distribution of the parasite. 

Concluding remarks

According to the study in Spain, the presence of *A. cantonensis,* not only in rats from the sewer system but also from city parks and orchards located in the peri-urban area of Valencia, shows a progressive invasion of internal territories by the parasite; however, its presence in rats does not necessarily mean that the disease will become a relevant public health concern since its occurrence strongly depends on the food habits of the population at risk. Therefore, risk is minimal if proper precautions are taken. 

The *take-home* message from the two studies is that neuroangiostrongyliasis should be included in the differential diagnosis of eosinophilic meningitis, independently of the classical endemic area of origin of the patient.
